# RNase A Inhibits Formation of Neutrophil Extracellular Traps in Subarachnoid Hemorrhage

**DOI:** 10.3389/fphys.2021.724611

**Published:** 2021-09-16

**Authors:** Anton Früh, Katharina Tielking, Felix Schoknecht, Shuheng Liu, Ulf C. Schneider, Silvia Fischer, Peter Vajkoczy, Ran Xu

**Affiliations:** ^1^Department of Neurosurgery, Charité – Universitätsmedizin Berlin, Corporate Member of Freie Universität Berlin, Humboldt-Universität zu Berlin, and Berlin Institute of Health, Berlin, Germany; ^2^Department of Biochemistry, Giessen University, Giessen, Germany

**Keywords:** neutrophil extracellular traps, neutrophils, subarachnoid hemorrhage, hemorrhagic stroke, neuroinflammation, innate immune response, innate immune reaction

## Abstract

**Background:** Subarachnoid hemorrhage (SAH) caused by rupture of an intracranial aneurysm, is a life-threatening emergency that is associated with substantial morbidity and mortality. Emerging evidence suggests involvement of the innate immune response in secondary brain injury, and a potential role of neutrophil extracellular traps (NETs) for SAH-associated neuroinflammation. In this study, we investigated the spatiotemporal patterns of NETs in SAH and the potential role of the RNase A (the bovine equivalent to human RNase 1) application on NET burden.

**Methods:** A total number of *n*=81 male C57Bl/6 mice were operated utilizing a filament perforation model to induce SAH, and Sham operation was performed for the corresponding control groups. To confirm the bleeding and exclude stroke and intracerebral hemorrhage, the animals received MRI after 24h. Mice were treated with intravenous injection of RNase A (42μg/kg body weight) or saline solution for the control groups, respectively. Quadruple-immunofluorescence (IF) staining for cell nuclei (DAPI), F-actin (phalloidin), citrullinated H3, and neurons (NeuN) was analyzed by confocal imaging and used to quantify NET abundance in the subarachnoid space (SAS) and brain parenchyma. To quantify NETs in human SAH patients, cerebrospinal spinal fluid (CSF) and blood samples from day 1, 2, 7, and 14 after bleeding onset were analyzed for double-stranded DNA (dsDNA) *via* Sytox Green.

**Results:** Neutrophil extracellular traps are released upon subarachnoid hemorrhage in the SAS on the ipsilateral bleeding site 24h after ictus. Over time, NETs showed progressive increase in the parenchyma on both ipsi- and contralateral site, peaking on day 14 in periventricular localization. In CSF and blood samples of patients with aneurysmal SAH, NETs also increased gradually over time with a peak on day 7. RNase application significantly reduced NET accumulation in basal, cortical, and periventricular areas.

**Conclusion:** Neutrophil extracellular trap formation following SAH originates in the ipsilateral SAS of the bleeding site and spreads gradually over time to basal, cortical, and periventricular areas in the parenchyma within 14days. Intravenous RNase application abrogates NET burden significantly in the brain parenchyma, underpinning a potential role in modulation of the innate immune activation after SAH.

## Introduction

Subarachnoid hemorrhage (SAH) caused by rupture of an intracranial aneurysm, is a life-threatening emergency that is associated with substantial morbidity and mortality (Lawton and Vates, 2017; Van Lieshout et al., 2018). Approximately 5% of all strokes are caused by SAH and the incidence is estimated to be nine per 100.000 person years (Etminan et al., 2019). Women are about 1.24 times more likely to be affected by the disease, and the incidence of SAH increases from the age>50years and is higher in Japan and Finland compared to other industrialized countries (Macdonald and Schweizer, 2017). Around one-third of SAH patients die within the first 30days after the initial bleeding event (Schertz et al., 2016). Among survivors, secondary brain injury is described as the main cause of morbidity (Macdonald, 2014). Thereby, emerging evidence points toward involvement of inflammatory processes (Gris et al., 2019), particularly of innate immune cells (Schneider et al., 2015; Lucke-Wold et al., 2016; Zhang et al., 2020).

Among these immune reactions, recent studies suggest that neutrophils play a particular role in SAH induced inflammation (Atangana et al., 2017). Neutrophil granulocytes are the first line of the innate immune system to fight pathogens (Borregaard, 2010). During infectious processes, they exit blood vessel systems, migrate to the site of infection and accumulate in high numbers (Niemiec et al., 2015). Neutrophil infiltration into damaged brain tissues has been shown after SAH, stroke, and traumatic brain injury (Liu et al., 2018; García-Culebras et al., 2019; Zeng et al., 2021). Upon stimulation, neutrophils release DNA, granule proteins, and histones (Brinkmann et al., 2004). These fibril matrixes are coined as neutrophil extracellular traps (NETs). NETs are involved in various immune reactions and the pathophysiology of diverse diseases (Papayannopoulos, 2018), including central nervous system pathologies (Zhang et al., 2020). The impact of NET formation in SAH has not yet been studied extensively, but recent evidence suggests that they may promote aneurysm rupture, and pharmacological removal of NETs can reduce the rate of aneurysm rupture (Korai et al., 2021). Moreover, there is growing evidence that NETs aggravate the inflammatory events after SAH, and impair revascularization and increase blood brain barrier (BBB) damage after stroke (Kang et al., 2020).

Next to extracellular DNA, other alarmins or so-called danger-associated molecular pattern (DAMP) signals such as extracellular RNA (exRNA) have been described as a key player in the involvement of central system pathologies, including hemorrhagic stroke (Tielking et al., 2019). In this context, RNase is a natural enzyme involved in the regulation of vascular homeostasis that can counteract exRNA when applied exogenously (Fischer et al., 2011; Lasch et al., 2020; Preissner et al., 2020). Recent studies have shown that NET-associated RNA is a relevant NET component and its formation can occur also independently of DNA, which opened the hypothetical avenue that RNase A (the bovine equivalent to human RNAse1) may also modify NET formation *in vivo* SAH models (Herster et al., 2020). Moreover, preliminary data from our laboratory show that RNase A modulates exRNA accumulation in SAH and the microglia-specific immune reaction after SAH.

In this study, with regard to the migratory characteristics of neutrophils, we investigated the spatial and temporal pattern of NET formation in an *in vivo* model of SAH. We report that NET accumulation begins in the ipsilateral side of bleeding, and spreads over time to the parenchyma on both hemispheres, peaking on day 14 after SAH. To confirm these findings in the human system, we also measured NET burden in cerebrospinal spinal fluid (CSF) and blood samples in SAH patients and show that NET accumulation occurs in both compartments significantly. Furthermore, we report that RNase significantly reduces NET formation in the parenchyma, thus being an attractive mediator for evaluation in subsequent studies.

## Materials and Methods

### Study Approval

The analysis on human samples was approved by the local ethics committee of Charité University Hospital (ethical approval number: EA2/134/18). All patients or their authorized individuals gave written consent to the collection of blood and CSF samples. All animal experiments were approved by the Regional Office for Health and Social Affairs (Landesamt für Gesundheit und Soziales; approval number: TVA 0063/18) and were performed in conformity with the German law of animal protection and the National Institute of Health Guidelines for care and use of laboratory animals.

### Human Data

#### Measurement of NET Surrogate Markers in CSF and Peripheral Blood in SAH Patients

Peripheral blood and CSF samples were collected from patients with aneurysmal SAH on day 1, 2, 7, and 14 after SAH onset. For each time point, 2ml peripheral blood was drawn using Ethylene Diaminetetraacetic acid (EDTA) tubes, and 2ml CSF was collected from the initial placement of an extraventricular drain (EVD) at onset of SAH, and then further collected from the existing EVD. Some patients also had additional lumbar drainage, but in this study only ventricular CSF was used. Blood and CSF samples were immediately placed on ice and spun down twice at 4°C at 500g for 5min, and supernatant was frozen at −80°C before further analysis. Double-stranded DNA (dsDNA) was quantified in CSF and plasma samples as previously published (Ondracek et al., 2020). In detail, samples were incubated with Sytox Green (Thermo Fisher), a fluorescent dsDNA-binding dye, in a concentration of 1μM for 5min. Fluorescence intensities (excitation 480nm, emission 520nm) were measured in 96-well microplates using a Tecan Infinite M200 reader. Values were normalized to a standard curve of dsDNA (Lambda DNA, Thermo Fisher).

### Animal Experiments

Male C57BL/6-mice were kept at the animal facility at the Neuroscience Research Center at Charité University (NWFZ, Berlin). Their age ranged from 8 to 12weeks and with a weight range of 18–28g. Animals underwent SAH operation, and sham operation (as the control condition), and were sacrificed at three different time points (1, 7, and 14days) after operation. A total number of *n*=81 mice were included in this study.

#### Mouse Model of Subarachnoid Hemorrhage

Subarachnoid hemorrhage was induced with a filament perforation model as described previously (Schneider et al., 2015). Briefly, mice were anesthetized with an intraperitoneal ketamine/xylazine (70mg resp. 16mg/kg body weight) injection and placed in supine position. Starting with a midline neck incision, the carotid artery was exposed and a 5–0 non-absorbable monofilament polypropylene suture inserted into the external carotid artery in a retrograde manner and advanced into the common carotid artery. In a next step, the filament invaginated into the internal carotid artery (ICA) and pushed forward to perforate the intracranial arterial bifurcation. Mice were perfused intracardially with PBS.

#### MRI

**Figure 1:**
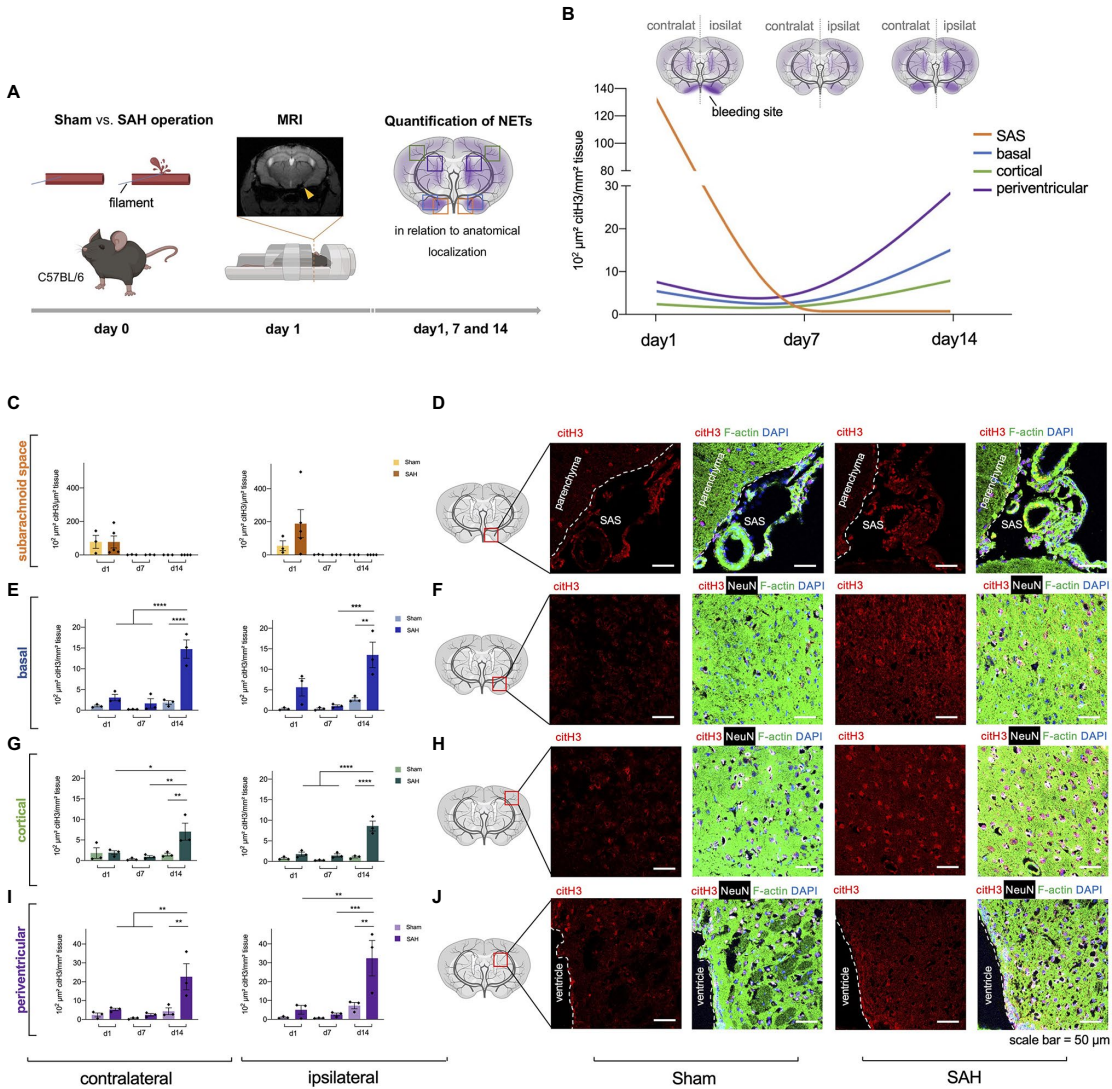
Neutrophil extracellular traps (NETs) are increased in subarachnoid hemorrhage (SAH). **(A)** The filament perforation model was utilized to induce SAH in C57BL/6 mice. MRI was done 24h after SAH onset to verify bleeding and exclude stroke or intracerebral hemorrhage, and NETs were quantified *via* immunofluorescence in relation to anatomical localization on three different time points (day 1, 7, and 14). **(B)** Cumulative data of NETs accumulation in SAH condition over time. **(C,D)** Quantification of NETs in subarachnoid space (SAS) with representative immunofluorescence staining of extracellular citH3 (exCitH3), F-actin, NeuN, and DAPI. Sham, *n*=3 for each time point; SAH, *n*=5 for each time point. **(E–J)** Quantification of NETs in basal, cortical, and periventricular parenchyma with representative immunofluorescence staining of citH3 (citrullinated H3), F-actin, NeuN, and DAPI on day 1, 7, and 14. Sham, *n*=3 for each time point; SAH, *n*=3 for each time point. contralat., contralateral; ipsilat., ipsilateral. Scale bar=50μm. ^*^ *p*<0.05, ^**^ *p*<0.01, ^***^ *p*<0.001, and ^****^ *p*<0.0001.

All SAH mice received an MRI 24h after surgery to confirm the bleeding and animals who had a stroke or intracranial hemorrhage (ICH) were excluded from the experiments (Figure 1A). The 1H magnetic resonance imaging was performed on a PharmaScan 70/16U (Bruker Corporation) with a field strength of 7Tesla by using the software Paravision 5.1 (Bruker Corporation). During the scans, mice received an O_2_/N_2_O+isoflurane gas anesthesia. The animals’ respiration was observed with Small Animal Monitoring System (SA Instruments, Inc.), while their temperature was maintained *via* controlled warming blankets. The presence of SAH was validated from T2 weighted image. Here, SAH volume was estimated based on the formula $V=\left(A_{1}+A_{2}+\ldots +A_{x}\right)\cdot d$, by which A_n_ corresponds to the bleeding area on each coronal MRI and d corresponds to the MRI slide thickness.

#### RNase Treatment

Both groups (Sham and SAH animals) received treatment with RNase A (Thermoscientific EN0531), which was administered intravenously (42μg/kg body weight) and intravenous application of saline solution was used as a control, as described previously (Walberer et al., 2009). The first injection was administered perioperatively to ensure penetration to the CNS due to breakdown of the BBB after SAH surgery. Due to anesthesia of mice during operation as well their supine positioning, the first injection was applied *via* the sublingual vein. The further injections were repeated every 3days in the tail vein until scarification as previously reported (Fischer et al., 2013). The total volume of injection was 100μl at a flow rate of 20μl/s.

#### Preparation of the Subarachnoid Space for Immunohistochemistry

To preserve the SAS, whole skulls were harvested and kept in an ascending concentration series of sucrose for proper dehydration (4days at 20%, 4days at 30%, and 4days at 40%) at 4°C and then snap frozen with isopentane. The skulls were then embedded in Tissue-Tek O.C.T. Compound (4583, Sakura Finetek) and carefully cut into 10μm thin slices with Microm Sec35 blades (Termo APD Consumables) using a cryostat (Thermo Fisher Scientific Inc. Microm HM 560).

### Immunofluorescence

Brain sections were blocked for 30min with 5% bovine serum albumin (BSA) in Tris-Buffered Saline with 0.05% Tween (TBST) at room temperature. After that, primary antibodies against citrullinated H3 (rabbit, 1:30, ab5103, Abcam), NeuN (rabbit, 1:200, ABN78, Millipore), and F-actin (Alexa Fluor 488 Phalloidin, 1:200, A12379, Thermo Fisher Scientific) diluted in 5% BSA/TBST were added for incubation at 4°C over night. The next day, sections were washed three times in 5% BSA/TBST for 10min, followed by 1.5 h of incubation with the secondary antibodies Rhodamine Red-X-conjugated Donkey IgG anti-Mouse (1:200, 715-295-151, Dianova) and Alexa Fluor 647-conjugated Donkey IgG anti-Rabbit IgG (1:200, 711-605-152, Dianova), diluted in 5% BSA/TBST. After secondary antibody incubation, specimens were washed three times in PBS for 5min and mounted with DAPI Mounting Medium (SCR-038448, Dianova). The sections were then imaged with confocal microscopy (Leica DM 6000/SP5) and analyzed with ImageJ. Amount of NETs was determined by calculating the ratio area covered by citH3 over total area covered by phalloidin, NeuN, and DAPI (Supplementary Figure S1).

### Statistical Analysis and Figures

**Figure 2:**
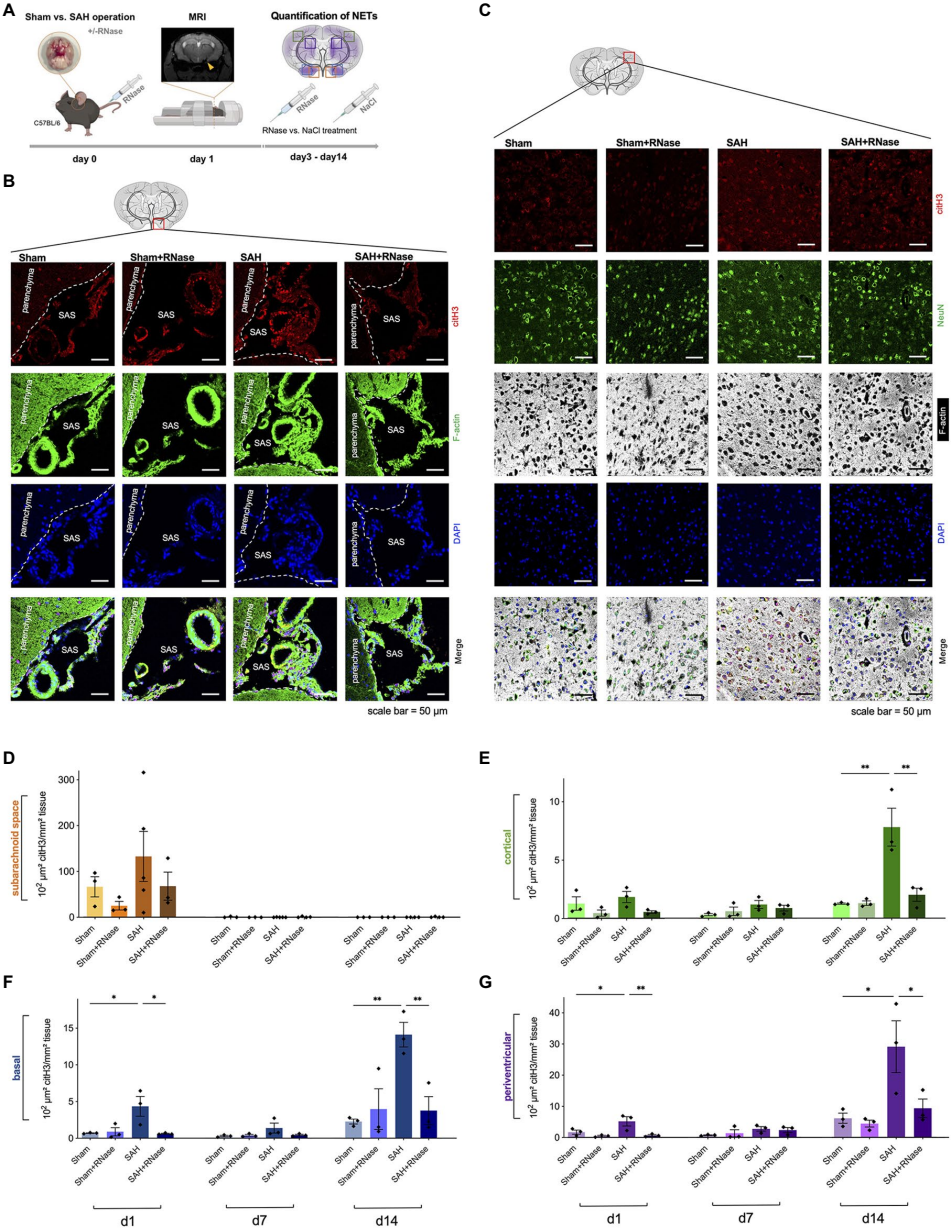
RNase A treatment reduces accumulation of NETs. **(A)** Experimental setup for RNase treatment: during operative induction of SAH, mice were treated with RNase A (42μg/kg body weight) or sodium chloride (NaCl) as a control condition, and MRI to confirm bleeding. RNase A treatment was repeated every 3days. **(B)** Representative images of triple immunofluorescence staining with citH3, F-actin, and DAPI subarachnoid space of the four subgroups (Sham, *n*=3; Sham+RNase, *n*=3; SAH, *n*=3; and SAH+RNase, *n*=3) on day 1, scale bar=50μm. **(C)** Representative images of quadruple immunofluorescence staining with citH3, NeuN, F-actin, and DAPI of the corresponding four subgroups in cortical area on day 14, scale bar=50μm. **(D–G)** Quantification of NETs after RNase A treatment in relation to localization (subarachnoid space, basal, cortical, and periventricular parenchyma) and time course (day 1, 7, and 14). SAS, subarachnoid space. ^*^ *p*<0.05 and ^**^ *p*<0.01.

**Figure 3:**
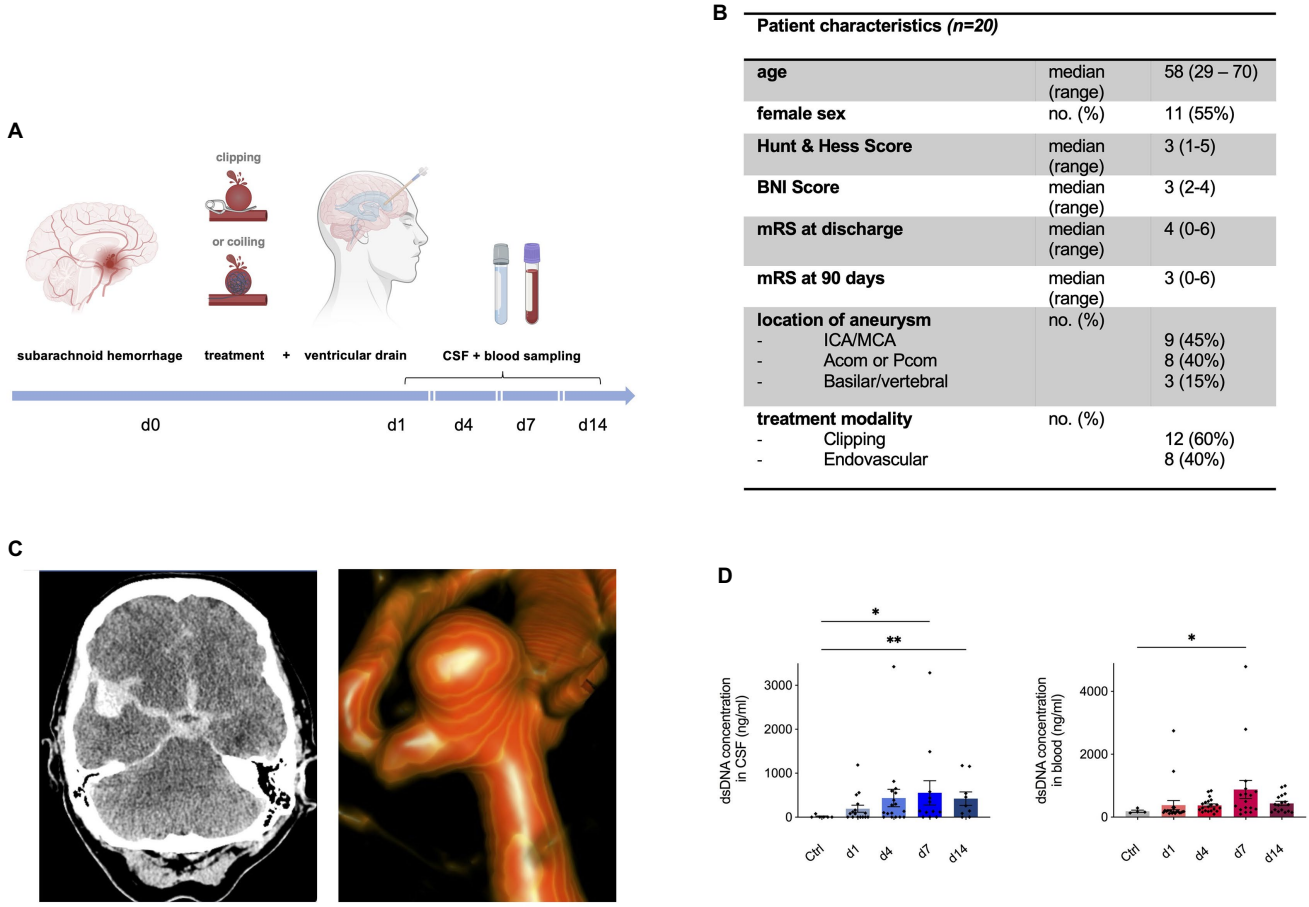
Neutrophil extracellular traps are increased in human cerebrospinal spinal fluid (CSF) and peripheral blood after aneurysmal SAH. **(A)** Study protocol for collection of CSF and Ethylene Diaminetetraacetic acid (EDTA) blood; patients with aneurysmal SAH received either microsurgical clipping or endovascular intervention (coiling) with placement of an extraventricular drain (EVD). In the course of 14days, CSF and peripheral blood were collected in a prospective manner. **(B)** Overview of patient characteristics. **(C)** Illustrative cases of SAH. CT imaging shows hyperdense blood collection in subarachnoid space and in the right sylvian fissure of a 55-year old patient who experienced SAH from a right-sided MCA aneurysm who underwent microsurgical clipping (left). 3D reconstruction of angiogram showing an ACom aneurysm of a 59-year old patient who was further treated *via* coiling. **(D)** Double-stranded DNA (dsDNA) concentration in CSF and peripheral blood of control patients and SAH patients shows peak on day 7 after bleeding onset. Acom, anterior communicating artery; BNI score, barrow neurological institute score; CSF, cerebrospinal fluid; ICA, internal carotid artery; MCA, middle cerebral artery; mRs, modified Rankin score; and Pcom, posterior communicating artery. ^*^ *p*<0.05 and ^**^ *p*<0.01.

Data were analyzed using GraphPad Prism for statistical analyses (Graphpad Software, Version 6.0). ANOVA analyses were used to compare multiple, unpaired *t*-tests for the comparison of two groups. The values are displayed as means±SEs and values of *p*<0.05 were considered statistically significant. Elements of Figures 1A, 2A,B, 3A were composed with BioRender.com.

## Results

### NETs Are Released After Onset of Subarachnoid Bleeding in an *in vivo* Mouse Model of Subarachnoid Hemorrhage

To determine whether NETs are released upon subarachnoid hemorrhage *in vivo*, we utilized a filament perforation model to induce subarachnoid hemorrhage in C57BL/6 mice, while Sham operation was performed as a control condition. MRI was conducted 24h after bleeding to verify SAH and exclude other pathologies such as stroke or intracerebral hemorrhage (Figure 1A), and NETs quantified *via* immunofluorescence staining using citrullinated Histone (citH3), a known specific marker for NET remnants (Wang et al., 2009). Indeed, increased levels of NET formation were found in SAH with the highest amount in the SAS on the ipsilateral side (where bleeding was induced) on day 1 (Figures 1C,D), while after this time point they were essentially not detectable anymore in both control and SAH conditions (Figure 1C). In contrast, in the parenchyma, the NETs showed a gradual increase over time with the highest density on day 14 in all localizations (Figures 1E–J). Interestingly, NET accumulation followed a spreading pattern within the parenchyma from basal to cortical over time, with the most significant increase in periventricular localization (Figure 1B).

### Pharmacological Modulation With RNase A Reduces NET Formation

Since recent evidence suggests that NET-associated RNA is a physiologically relevant NET component and its formation can occur also independently of the canonical NET component DNA (Herster et al., 2020), we questioned whether RNase A application can modify NET burden in SAH *in vivo*. RNase A seemed a feasible therapeutic approach since previous studies in our group showed that RNase A can modulate other DAMPs including exRNA, and influence the microglia-specific immune reaction after SAH. Hence, we treated mice with intravenous application of RNase A and quantified NETs in both SAS and parenchyma (Figure 2A). In SAS, RNase A decreased NET accumulation on day 1, but this was not statistically significant. In contrast, in the parenchyma, RNase A reduced NET accumulation significantly, specifically on day 14, where accumulation of citH3 peaked in all localizations (Figures 2B–G).

### NETs Are Increased in Both CSF and Blood of SAH Patients

Next, we questioned whether this observed NET accumulation after SAH is also relevant in the human system. To investigate this, CSF and blood samples were collected within the scope of a prospective observational study in aneurysmal SAH patients (Figure 3A), and NETs were measured *via* quantification of dsDNA *via* Sytox Green. Figure 3B shows the baseline characteristics of the SAH patients included in the study. Figure 3C illustrates imaging from two exemplary cases of SAH patients: on the left side, a CT scan of a 55-year old female patient is shown with SAH (Hunt&Hess grade II) with a ruptured right-sided middle cerebral artery (MCA) aneurysm who underwent microsurgical clipping; on the right side, a three-dimensional reconstruction of a ruptured anterior communicating artery (ACom) aneurysm of a 59-year old female (Hunt & Hess grade II) is shown who was further treated with aneurysm coiling. The control patients comprised of patients in which lumbar puncture was done to rule out meningitis or SAH without evidence of any intracranial pathology. Interestingly, dsDNA was significantly increased in both CSF and peripheral blood of SAH patients compared to the control group, with a gradual increase over time, peaking in both compartments on day 7 (Figure 3D).

## Discussion

Aneurysmal SAH remains a devastating pathology with high morbidity and mortality, and attempts to reduce secondary brain damage have been made for decades, yet the exact pathomechanism contributing to the long-term damage is unclear (Black, 1986; Peterson et al., 1990; Mathiesen et al., 1997; Sheehan et al., 1999). Recent data suggests involvement of the immune system attributable to secondary brain damage, specifically through an outside-in activation of neutrophil recruitment to endothelium, contributing to microglia activation and neuronal apoptosis (Schneider et al., 2015; Atangana et al., 2017). Among the classical immune defense mechanisms of neutrophils, consisting of engulfment of microbes and secretion of anti-microbials, recent data pinpoints to a novel function of neutrophils as part of the innate immune response – the formation of NETs to kill extracellular pathogens (Brinkmann et al., 2004).

In this study, we sought to investigate the potential role of NET formation in SAH and describe the spatiotemporal patterns of NET accumulation after SAH. We demonstrate that in the acute setting, a direct flood of NET formation occurs in the ipsilateral subarachnoid space (SAS), while in the parenchyma, NET levels increase gradually over time in basal, cortical, and periventricular compartments distant to the region of bleeding. These findings are supported by a recent study reporting NET accumulation after SAH as well as their involvement in neuroinflammatory events, albeit the time point of the peak of NETs differed to some degree (Zeng et al., 2021). Interestingly, in the study by Zeng et al. (2021), inhibition of NET formation *via* the PAD4 antagonist GSK484 as well as DNase I inhibited NET-associated neuroinflammation. Our data show that the quantitatively most relevant NET formation occurred 14days after SAH specifically in periventricular localizations. This is intriguing as it raises the question whether NETs may also be involved in CSF hydrodynamics after aneurysmal SAH, especially since post-hemorrhagic hydrocephalus is a common complication after SAH (Germanwala et al., 2010).

Moreover, while a direct inhibition of NETs *via* DNase has been postulated as a potential mechanistic treatment strategy to reduce NET burden (Ondracek et al., 2020; Korai et al., 2021), there is mounting evidence that NET-associated RNA is a relevant component of NET formation and can occur independently of DNA (Herster et al., 2020). Based on this finding and previously published data on the dampening effects of RNase A on immune cells (Lasch et al., 2020), we questioned whether RNase A also modifies NET formation. Indeed, our data show that RNase A significantly reduces NET formation in all compartments of the brain. This specific effect on the CNS may be explained by the blood brain barrier breakdown after SAH with permeability to also bigger proteins such as Evans Blue (70kDa; Blecharz-Lang et al., 2018). In our experiments, we used a 13.7kDa pancreatic RNase A for pre- and postoperative intravenous injections. These finding are particular compelling since RNAse therapeutics have already been used to some degree in clinical trials (Mikulski et al., 2002; Ardelt et al., 2007; Chang et al., 2010; Squiquera et al., 2017). Therefore, it is tempting to speculate that RNAse may be an accessible interesting potential therapeutic strategy for treatment of the underlying immune reaction after SAH. However, the influence of i.v. application of RNAse on physiological processes such as regulation of cerebral blood flow was not investigated in this study and should be addressed in further studies.

The exact molecular mechanisms of NET formation are not fully understood. SAH promotes generation of reactive oxygen species (ROS; Ayer and Zhang, 2008), that have also been described as triggers of NET formation (Erpenbeck and Schön, 2017). Therefore, one may pose the hypothesis that ROS-activation in the setting of SAH contributes NET accumulation, which then may promote microglial activation leading to the activation of neuroinflammatory cascades. Furthermore, recent studies have described that NET formation in the context of SAH increases levels of cytokines such as IL-1β, IL-6, and TNF-α (Zeng et al., 2021).

Furthermore, in order to investigate whether NET burden is also relevant in the human system, we measured circulating NET abundance *via* the surrogate marker dsDNA and show that increased levels are found in both CSF and peripheral blood of SAH patients. Here, we observed a peak of dsDNA 7days after the bleeding event, supporting the *in vivo* data from our SAH mouse model. Our findings are in line a recent study of Zeng et al. (2021) showed that a significant increase of citH3 in patients suffering SAH after 24h of the bleeding event, which correlated with the clinical Hunt and Hess score. Additionally, we showed for the first time the further temporal dynamics of NETs and their involvement in CSF in humans. The postponed peak of NET burden potentially indicates a therapeutic window after the bleeding event to attenuate secondary brain damage after SAH. The accumulation of NETs in the brain parenchyma also raises intriguing questions of its origin. Increasing levels of neutrophils in the CSF after SAH have also been associated with development of vasospasms (Provencio et al., 2010). Therefore, neutrophils may migrate in the brain parenchyma after SAH and produce NETs *in situ* as already described in other studies in a model of ischemic stroke (Kang et al., 2020).

Our study is limited by a small sample size, investigations on the association of NET burden and clinical characteristics, and exploratory studies on cell-specific mechanism by which NETs may affect further inflammatory events in SAH. Albeit the range of the SAH patients in our small study cohort varies between 29 and 70, the median age of our study group in the patients is 58, which is fairly representative of typical age of aneurysm rupture in large cohorts (Rinkel et al., 1998; Shea et al., 2007; Jordan et al., 2009). In order to investigate potential aging effects, further subgroup analyses on young and old mice with larger study numbers are necessary. As we analyzed exclusively male mice, sex-related effects in the animal experiments cannot be excluded. This study also did not investigate the impact of RNAse treatment on humans. As potential neuroprotective effects of RNase in SAH patients are of pressing interest, a clinical study on the effect of RNase treatment in hemorrhagic stroke patients are planned for the future.

In summary, our data reveal the spatiotemporal dynamics of NET accumulation after SAH *in vivo* with evidence for a gradual increase of NET formation over time, both in a SAH mouse model as well as in patients suffering aneurysmal SAH. Intriguingly, intravenous RNase A application abrogates NET burden in the parenchyma, underpinning a potential role in of RNase in the innate immune response after SAH. Further studies are needed to fully elucidate the exact nature of NET formation and related immune cell-specific changes of RNase application after SAH.

## Data Availability Statement

The raw data supporting the conclusions of this article will be made available by the authors, without undue reservation.

## Ethics Statement

The studies involving human participants were reviewed and approved by Ethikkommission Charité, Charité – Universitätsmedizin, Berlin (Germany). The patients/participants provided their written informed consent to participate in this study. The animal study was reviewed and approved by Landesamt für Gesundheit und Soziales (LaGeSo) Berlin (Germany).

## Author Contributions

KT, FS, AF, SL, and RX conducted the experiments and analyzed the data. RX, AF, and PV designed the study. All authors contributed to the article and approved the submitted version.

## Funding

RX was supported by the BIH-Charité Clinician Scientist Program funded by the Charité – Universitätsmedizin Berlin and the Berlin Institute of Health. We acknowledge support from the German Research Foundation (DFG) and the Open Access Publication Fund of Charité – Universitätsmedizin Berlin. KT is supported by the Berlin Institute of Health (BIH) Research Stipend as well as the Sonnenfeld Foundation.

## Conflict of Interest

The authors declare that the research was conducted in the absence of any commercial or financial relationships that could be construed as a potential conflict of interest.

## Publisher’s Note

All claims expressed in this article are solely those of the authors and do not necessarily represent those of their affiliated organizations, or those of the publisher, the editors and the reviewers. Any product that may be evaluated in this article, or claim that may be made by its manufacturer, is not guaranteed or endorsed by the publisher.
