# Supplementary material for: RNase A Inhibits Formation of Neutrophil Extracellular Traps in Subarachnoid Hemorrhage
Source: Front Physiol. 2021 Sep 16;12:724611. doi: 10.3389/fphys.2021.724611 (PMC8481772; doi:10.3389/fphys.2021.724611)
Supplement: Supplementary file 1 [file Image_1.pdf]

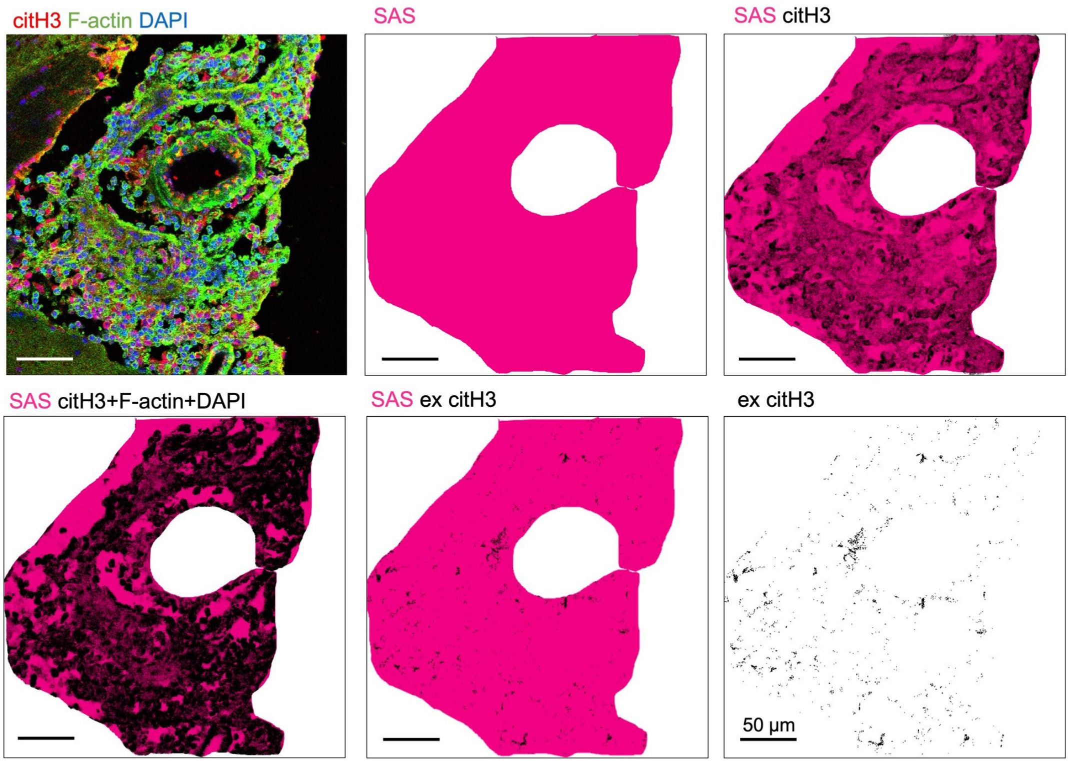

$$\mathbf{A}_{\mathbf{exCitH3(SAS)}} = \frac{\mathbf{A}_{\mathbf{H3}} - \mathbf{A}_{\mathbf{F-actin+citH3+DAPI}}}{\mathbf{A}_{\mathbf{SAS}}} = \frac{\mathbf{A}_{\mathbf{exCitH3}}}{\mathbf{A}_{\mathbf{SAS}}}$$

Supplementary figure 1. Quantification of exCitH3 (extracellular citrullinated H3) in subarachnoid space (SAS). Triple immunofluorescence staining of citH3 (citrullinated H3), F-actin, and DAPI. Area of exCitH3 was calculated based on the formula $\mathbf{A}_{\mathbf{exCitH3(SAS)}} = \frac{\mathbf{A}_{\mathbf{H3}} - \mathbf{A}_{\mathbf{F-actin+citH3+DAPI}}}{\mathbf{A}_{\mathbf{SAS}}} = \frac{\mathbf{A}_{\mathbf{exCitH3}}}{\mathbf{A}_{\mathbf{SAS}}}$. Scale bar = 50 μm.
